# Attitudes toward active aging and their association with social determinants and views on older adults in Japan: a cross-sectional study

**DOI:** 10.1186/s12877-024-04711-0

**Published:** 2024-02-07

**Authors:** Eri Osawa, Yuri Sasaki, Hui-Chuan Hsu, Hiroko Miura

**Affiliations:** 1 Department of Public Health Policy, National Institute of Public Health, 351-0197 Saitama, Japan; 2 School of Public Health, Research Centre of Health Equity, College of Public Health, Taipei Medical University, 11031 Taipei, Taiwan; 3 Division of Disease Control and Epidemiology, School of Dentistry, Health Sciences University of Hokkaido, 061-0293 Tobetsu, Hokkaido Japan

**Keywords:** Active aging, Attitude, Views on older adults, Japan

## Abstract

**Background:**

Globally, the population of older adults has greatly increased, and active aging—whereby older adults can live healthy and fulfilling lives—is considered crucial for a sustainable society. However, the concept and practice of active aging are highly debated because it is unclear how people perceive active aging. This study explored Japanese people’s attitudes toward active aging (ATAA) and examined the associations between ATAA scores and sociodemographic variables, views on older adults, and self-rated life and health.

**Methods:**

This study used data obtained from an online survey that originally targeted adults of all generations in Taiwan, South Korea, and Japan. In this study, we used only data from Japanese participants to elaborate on factors associated with ATAA in Japan. We conducted a one-way analysis of variance test and multiple linear regression analysis to evaluate the associations between the ATAA scores of 506 Japanese individuals and sociodemographic variables, views on older adults, and self-rated life and health.

**Results:**

The sample comprised 171 females and 335 males. The mean (± SD) ATAA score of the 506 respondents was 138.8 (± 20.80). Females had a significantly higher ATAA score than males (144.02 versus 136.13, F = 26.29, *p* < 0.001). The respondents with higher education attainment, religious beliefs, better views on older adults, and better self-rated health were more likely to have a positive ATAA score (B: 3.83, 95% CI: 0.11, 7.56; B: 4.31, 95% CI: 0.93, 7.69; B: 2.07, 95% CI: 1.61, 2.53; B: 2.87, 95% CI: 0.92, 4.82, respectively). Being male, single (i.e., never married, divorced, or widowed) and other non-married marital statuses, and satisfied with one’s financial condition were negatively associated with ATAA (B: -8.73, 95% CI: -12.49, -4.96; B: -5.47, 95% CI: -9.07, -1.86; B: -2.04, 95% CI: -3.99, -0.09, respectively).

**Conclusions:**

This study identified that females have more positive ATAA than males. Better views on older adults are a possible contributing factor that promotes ATAA among Japanese people. Our findings provide useful evidence that an approach towards those who are male, single, and economically satisfied is needed so that they have a positive attitude toward aging in Japan. It is necessary to address ageism and develop an environment in which individuals can expect to age actively.

**Supplementary Information:**

The online version contains supplementary material available at 10.1186/s12877-024-04711-0.

## Background

Aging positively and healthily has always been a central theme of discussion in Japan, a super-aged society. However, in Japan, approximately 40% of older adults suffer from loneliness [1], and the number of socially isolated individuals has been increasing [2]. Various types of interventions have been reported and evaluated to help older adults lead an active and healthy life in Japan [3–6].

Active aging has become a paradigm for ensuring a sustainable environment for a world with an aging population. It has played an increasingly significant role not only in research but also in policy development and implementation [7]. There is a plethora of terms expressing “aging well,” such as active aging, healthy aging, productive aging, and successful aging. Successful aging was initially developed as a research tool to establish an intellectual and methodological foundation for interdisciplinary gerontology that emphasizes the importance of individual, societal, and scientific conceptualizations and understandings of aging [8], and it is a term that has been employed in the United States. It is based on the concept that later life experiences can be considered in terms of success, rather than conventional expectations of loss and decline caused by aging [9, 10].

The concept of active aging was proposed in Europe in the 1990s. It emphasizes encouraging the participation of older adults in society, as well as increasing their competence and knowledge. The framework of active aging also appeals to policy decisions [10]. The World Health Organization (WHO) defines active aging as “the process of optimizing opportunities for health, participation, and security to enhance the quality of life as people age” [11]. The concept of active aging is broader and more multidimensional than healthy aging and productive aging [12, 13]. Active aging has a positive effect on life satisfaction, and active coping styles are associated with a higher possibility of active aging [7]. To evaluate the enabling social environment, the Active Aging Index (AAI) was developed with 22 indicators categorized into four groups: employment; participation in society; independence, health, and secure living; capacity and an enabling environment [14]. The European Commission and the United Nations Economic Commission for Europe have used the AAI to assess the active aging status of European countries and have provided suggestions for policymakers since its introduction [15, 16]. Recently, several Asian countries have started to apply the AAI to examine their countries’ active aging status and use it for policymaking [17–19].

As the world’s population ages, it is worth examining how a positive life in old age can be obtained. However, to date, attitudes toward aging have mainly been explored in terms of its negative aspects, such as ageism. Most studies of ageism have focused on its effects and severe consequences in older adults [20, 21]. Several theories explain the manifestations of ageism. For example, modern capitalist economies have marginalized older people into enforced retirement, thus lowering their economic and social status and promoting the assumption that older people are unproductive and contribute little to society [22]. Additionally, ageism is explained by psychological models such as the self-categorization theory, which reflects the psychological use of age category boundaries for sharing views and interests [23], and the intergroup threat theory, which describes the social perception that older adults are a burden on healthcare and welfare resources [24].

There are also arguments regarding the concept of active aging and its uncertainty in practice. One such argument is that the AAI that emerged in Western cultures may not fit Asian countries [25]. Although Asian cultures emphasize respect for older adults, negative personal attitudes toward older adults have begun to emerge [26]. The AAI focuses only on the outcomes and investment in the later life of older adults, while cumulative risks or investment from childhood or early adulthood may be underestimated [17]. Early investment in individuals and society would increase the potential effects of active aging [27].

The literature shows socio-economic factors such as gender, education, employment, and income as indicators of active aging. Previous studies provide evidence of gender differences in active aging. Gender differences in physical health among older adults have been found in multimorbidity [28], healthy life expectancy, and physical function [29, 30]. Education has also been associated with both healthy and risky behaviors [31, 32] and social participation [33]. Barslund et al. showed large differences in the distribution of individual-level active aging across age groups in the 13 European countries they covered [34]. Our previous study [25] using pooled data collected from three Asian countries (Taiwan, South Korea, and Japan) found that Japanese people generally had poorer attitudes toward active aging (ATAA) and had a more negative view of older adults than people in Taiwan and Korea. The country differences in the ATAA reflect a country’s cultural and social situation. Therefore, the current study focused on only Japanese individuals and investigated associations between the total ATAA score and views on older adults, and social determinants such as gender, age, residential area, marital status, education, occupation, religious beliefs, and recognition of one’s life and health to understand Japanese comprehensive attitude toward aging. The original study, on the other hand, examined the association among five sub-components of ATAA and impression on and relationship with older adults and socio-demographics, mainly nationality and age, to identify different aspects of ATAA among three countries. This allowed for a detailed examination of factors among Japanese individuals that were not mentioned in the previous study comparing three countries. Specifically, this study aimed to (1) explore Japanese people’s ATAA and (2) examine the associations between ATAA and social determinants and views on older adults. We hypothesized that gender, age, educational background, and views on older adults would be highly associated with ATAA.

## Methods

### Data and study participants

The study used data from an online survey conducted by Survey Planet, LLC, which initially targeted adults of all generations in Taiwan, South Korea, and Japan during the summer of 2019. Five hundred and nine Japanese individuals aged 20 years or older responded to the survey, and 506 answered all of the questions (valid response rate = 99.4%). We analyzed these valid responses to examine ATAA in Japan.

### The ATAA scale

The ATAA scale was designed based on the AAI, which has 19 indicators related to employment, voluntary activities, caring for others, political participation, physical activities, access to health and dental care, and so on [35]. To ensure the reliability and validity of the questionnaire, a pilot study was conducted to develop a draft of the questionnaire in English, and the authors of the previous study examined and revised its English version to increase the face validity [25]. The English version of the questionnaire was translated into Japanese and validated by the authors, and the Japanese questionnaires were pre-tested before the formal survey. The scale has 37 items (Table 1) and asks, “How important would you rate XX [each item] when you reach the age of 65 or above?” Responses were provided using a Likert scale (1 = very unimportant; 5 = very important). The details of each item are described in the previous study [25]. The internal consistency of the 37 items on the ATAA scale was high, with a Cronbach’s alpha of 0.94. Although we did not analyze sub-components of ATAA in the current study, the ATAA scale was analyzed using principal factor analysis with an explained variance of 66.1% for seven factors (See supplementary Table 1).

**Table 1:** The 37 items comprising the ATAA score

Continuing to work until age 65
Continuing to work after age 65
Volunteering without a salary
Taking care of children in a family
Taking care of children in a community
Taking care of older adults or disabled individuals in a family
Taking care of older adults or disabled individuals in a community
Participating in political activity
Participating in social groups
Being independent in activiteis of daily living
Having a good memory
Being able to see a doctor convieniently and affordably
Feeling pleasant
Feeling calm
Feeling energetic
Doing physical activity or exercise regularly and frequently
Being able to use preventive health care
Owing assets
Owing enough house electric appliances
Being able to pay for utility bills
Having savings for at least 3-6 months living expenses
Having enough protein intake at least one meal per day
Not having to worry about being attacked at home or in the neighborhood
Not having to worry about transpotation accidents or injury in your living area
Having a chance to travel at least once a year
Having enough penstion for a living
Living with a family
Receiving mutual financial support with a family
Interacting with family or friends every week
Feeling trust toward a neighborhood/a community
Having an opportunity to receive informal education for lifelong learing
Using the internet
Going out in living area independently
Having no trouble in taking public transportation
Being barrier-free in public spaces
Having access to activities in a community that consider all-age participation
Feeling respected by society

### Independent variables

The independent variables included sociodemographic variables, views on older adults, and life and health variables. Sociodemographic variables, namely, age, area of residence, marital status, final education, occupational status, and religious beliefs, were treated as categorical sociodemographic variables. Views on older adults (65 years or older) included personal and social views. Personal view comprised five questions: (1) general impression of older adults, scored from 1 (negative) to 10 (positive); (2) willingness to live with an older family member, scored from 1 (negative) to 5 (positive); (3) willingness to work with older colleagues, scored from 1 to 5; (4) the contribution of older adults to their family in general, scored from 1 to 5; and (5) the contribution of older adults to society in general, scored from 1 to 5. The total score ranged from 5 to 30. The higher the score, the better the view of older adults. Social respect and inclusion of older adults were measured through the recognition of social respect and their inclusion in society, and were gauged using four questions: (1) social fairness to older adults, scored from 1 (unfair) to 5 (fair); (2) social respect for older adults, scored from 1 (no respect at all) to 6 (too much respect); (3) the inclusion of older adults in society by providing commodities tailored to their needs, scored from 1 (not at all) to 5 (provide all commodities); and (4) the inclusion of older adults by providing activities suitable for them, scored from 1 (not at all) to 5 (provide all). The total score ranged from 4 to 21. The higher the score, the greater the recognition of social respect and inclusion. For life and health variables, satisfaction with one’s financial condition, living condition, and self-rated health were reported on a scale ranging from 1 (very unsatisfied/unhealthy) to 5 (very satisfied/very healthy). Mental well-being was measured using the WHO’s five-well-being index (i.e., being calm, relaxed, energetic, refreshed, and filled with interesting things), reported on a scale ranging from 0 (*none of the time*) to 5 (*all the time*), with total scores ranging from 0 to 25.

### Statistical analysis

The mean ATAA scores for each sociodemographic variable were compared using a one-way analysis of variance (ANOVA) test as the ATAA scores were entered as continuous variables. Multiple linear regression analysis was performed to examine the associations of the respondents’ ATAA scores with the sociodemographic variables, views on older adults, and self-rated life and health. The adjusted multivariate results were expressed as non-standardized coefficients (B) and standardized coefficients (β) with 95% confidential intervals (CI). All analyses were conducted using STATA version 17 for Windows (Stata　Corp LLC, College Station, TX, USA).

### Ethical considerations

The study was conducted following the guidelines of the Declaration of Helsinki. The survey protocol was reviewed and approved by the TMU-Joint Institutional Review Board of Taipei Medical University (N201811051) and the Ethics Board of the National Institute of Public Health, Japan (NIPH-IBRA#12326). Voluntary participation and the right to withdraw from the study at any time were assured.

## Results

### ATAA scores by sociodemographic characteristics of respondents

Table 2 presents the ATAA scores for each sociodemographic variable. For the respondents’ characteristics, the mean (± SD) ATAA score was 138.8 (± 20.80). Females had a significantly higher ATAA score than males (144.02 versus 136.13, F = 26.29, *p* < 0.001). Similarly, marital status and religious beliefs were significantly different among the groups(at *p* < 0.05). The score for single (i.e., never married, divorced, or widowed) and other non-married individuals was lower than that for married individuals (134.69 versus 141.36, F:10.23, *p* < 0.05). Those with religious beliefs had a higher ATAA score than those without religious beliefs (141.34 versus 137.27, F = 7.51, *p* < 0.05).

**Table 2:** Attitude toward active aging (ATAA) scores by sociodemographic characteristics

Variables	n	mean	SD	F value*	p-value*
ATAA scores	506	138.8	20.80		
**Gender**					
Female	171	144.02	17.29		
Male	335	136.13	21.93	26.29	<0.001
**Age**					
20–39	148	139.76	23.98		
40–49	214	137.65	20.49		
60 and above	144	139.52	17.60	0.74	0.477
**Residential area**					
City	279	139.20	21.89		
Town or village	227	138.31	19.42	0.11	0.736
**Marital status**					
Married	312	141.36	17.74		
Single (never married, divorced, or widowed) and others	194	134.69	24.45	10.23	<0.05
**Highest education attained**					
High school or lower	144	135.14	22.32		
College, university, or higher	362	140.26	20.02	3.68	0.056
**Occupational status**					
Unemployed	150	138.18	20.09		
Part-time	74	136.46	24.31		
Full-time	282	139.74	20.19	2.46	0.087
**Have religious beliefs**					
No	316	137.27	21.04		
Yes	190	141.34	20.20	7.51	<0.05

SD = standard deviation

*One-way analysis of variance test was conducted; aR^2^ = 0.079

### Associations between ATAA and the sociodemographic variables, views on older adults, and life and health variables

Since multicollinearity was not observed among the independent variables, we used all variables in the multivariate analysis (variance inflation factor = 1.49). In the overall multivariate analysis (Table 3), males and single (i.e., never married, divorced, or widowed) and other non-married individuals were negatively associated with the ATAA (B: -8.73, 95% CI: -12.49, -4.96, B: -5.47, 95% CI: -9.07, -1.86, respectively). Graduation from college, university, or higher and having religious beliefs were positively associated with the ATAA (B: 3.83, 95% CI: 0.11, 7.56, B: 4.31, 95% CI: 0.93, 7.69, respectively) even after adjusting for other variables. Regarding views on older adults and life and health variables, views on older adults and self-rated health were positively associated with ATAA (B: 2.07, 95% CI: 1.61, 2.53; B: 2.87, 95% CI: 0.92, 4.82, respectively). By contrast, satisfaction with one’s financial condition was negatively associated with ATAA (B: -2.04, 95% CI: -3.99, -0.09). There was no interaction between gender and other sociodemographic variables according to the correlation coefficient among the independent variables (Supplementary Table 2) and the analysis with interaction terms between gender and other variables (Supplementary Table 3). The main effect of gender was found after we conducted regression analysis with interaction terms.

**Table 3:** Multivariate linear regression analysis between the total attitude toward active aging (ATAA) score and independent variables

Variables	ATAA (Total score)			
	ALL (*N* = 506)			
	95% CI			
	B	(β)	Lower	Upper
	aR^2^=0.233			
Intercept	107.13		95.77	118.50
**Sociodemographic variables**				
**Gender**				
Female	Ref			
Male	-8.73	(-0.20)	-12.49	-4.96
**Age**				
20–39	Ref			
40–49	-1.51	(-0.04)	-5.59	2.56
60 and above	-2.29	(-0.05)	-7.38	2.79
**Residential area**				
City	Ref			
Town or village	0.00	(0.00)	-3.26	3.26
**Marital status**				
Married	Ref			
Single (never married, divorced, or widowed) and others	-5.47	(-0.13)	-9.07	-1.86
Highest education attained				
High school or lower	Ref			
College, university, or higher	3.83	(0.08)	0.11	7.56
**Occupational status**				
Unemployed	Ref			
Part-time	-1.27	(-0.02)	-6.66	4.11
Full-time	2.55	(0.06)	-1.62	6.73
Have religious beliefs				
No	Ref			
Yes	4.31	(0.10)	0.93	7.69
**Recognition of older adults and one’s life and health**				
Views on older adults	2.07	(0.40)	1.61	2.53
Social respect and inclusion of older adults	-0.05	(-0.009)	-0.46	0.36
Satisfaction with financial condition	-2.04	(-0.11)	-3.99	-0.09
Satisfaction with living condition	0.42	(0.03)	-1.67	2.52
Self-rated health status	2.87	(0.13)	0.92	4.82
Mental well-being	-0.33	(-0.09)	-0.72	0.06

B = non-standadized coeffiecient, β = standadized coefficient, CI = confidential interval, aR2 = adjsted R-squared

The multivariate regression analysis was conducted between the total ATAA score and sociodemographic variables, views on older adults, and related factors

## Discussion

### The status of ATAA

This study explored ATAA and its associations with sociodemographic variables, views on older adults, and self-rated life and health in Japan. The study data indicated that the overall mean ATAA score was 138.8 (± 20.8), 144.02 (± 17.29) for females, and 136.13 (± 21.93) for males. Compared with the results of previous studies, which showed that females usually have a negative experience of active aging [36, 37], our results showed the opposite. ATAA may be considered as either an attitude or an expectation of one’s life in old age. A possible explanation of the contradictory results of the current study is that ATAA might differ from the evaluation of active aging in actual old age. In other words, there may be a gap between expectations (represented by attitudes) and the actual situation of active aging among females. Additionally, our previous study using pooled data in three countries [25] showed male have negative attitudes toward health and security, but positive attitudes toward social connection in ATAA. It is necessary to examine which aspects of the ATAA have differences between Japanese females and males as well.

In terms of marital status, being single (i.e., never married, divorced, or widowed) or otherwise not married showed a negative association with the ATAA. It is generally accepted that marriage positively contributes to one’s health and the aging process regardless of gender, thus becoming a protective factor against mortality [38]. Meanwhile, unmarried individuals living alone are at risk of early- and late-onset dementia [39]. For Japanese males, family-based social relationships have been associated with longevity [40]. Marriage may also affect one’s attitude toward aging. Our previous study targeting three countries [25] showed that having a spouse correlated with a positive attitude toward health and social participation but also a negative attitude toward social connection and work. Although a single status is not the only reason for a limited social connection, it is possible that having less social connection may be associated with negative attitudes toward aging. Graduation from college, university, or higher and having religious beliefs had a positive association with ATAA. Education, which is health- and life-related, might be also positively related to ATTA. Religion, spirituality, and beliefs play a role in the everyday lives of older adults, including being a source of strength, comfort, and hope during difficult times and bringing about a sense of community and belonging [41].

This study is unique in that it covered a wide age range: 20 to 86 years. Although no significant differences were observed among age groups, it is reasonable to assume that, in general, young versus old individuals differ in their attitudes toward old age [42, 43]. Additionally, generational differences must be considered when examining ATTA by age group [44]. This result was like the previous study among three countries [25]. The result of no significant association by age group may be due to a limitation of this study. Specifically, as this was an online survey, there may have been associations influenced by the characteristics of the participants, who were familiar with digital devices regardless of their age.

### Associations between ATAA and views on older adults and life and health variables

The results of this study showed that respondents with a better view of older adults were more likely to have a positive ATAA score. This result is similar to those of previous studies on ageism. Positive representations and intergenerational contact are the most important determinants of reducing ageism [45–47]. This finding suggests that one’s view of other older adults leads to a self-image of old age. Swift et al. [48] suggest that active aging strategies should recognize that age barriers and ageism need to be reduced.

A negative association between satisfaction with one’s financial condition and ATAA, and a positive association between self-rated health and ATAA were found in this study. Regarding the association between satisfaction with one’s financial condition, given that the survey did not ask about actual income, it was not possible to examine whether economic background is associated with ATAA. Although such an association is speculative, those who are economically well off may have poorer attitudes toward working and receiving pensions as they get older. Being healthy has an impact on active aging. Previous studies showed that not only physical health, but also self-rated health is an independent predictor of the decline of daily life function among older adults [49, 50], and that self-rated health in midlife has an association with active and healthy aging (e.g., through being alive and having no subjective cognitive problems in old age) [51]. Similarly, better self-rated health could support having a more positive ATAA.

### Limitations

This study had some limitations. First, owing to the online survey design, only those who were able to access the internet could respond to the questionnaire. Therefore, we could not eliminate selection bias, and the participants may not be representative of the Japanese population. Second, this was a cross-sectional study; therefore, causality could not be established. Third, the responses regarding living conditions were based on self-ratings. Therefore, the answers may not accurately represent actual living conditions. Despite these limitations, this study identified unique and novel factors related to the ATTA of Japanese individuals. Active aging measures that fit the Japanese context need to be further investigated and developed.

## Conclusions

This study identified the ATAA of Japanese people. It indicated that negative ATAA scores were associated with characteristics such as being male, having single-marital status, and being financially satisfied. Because there are differences in ATAA depending on certain sociodemographic characteristics, developing an environment in which the entire society can expect to age actively is required. Tackling ageism and creating a positive impression toward aging at a younger age should be prioritized when creating social institutions. This can help people develop a positive attitude toward aging.

As the world’s most aged society, Japan needs a sustainable society with active aging and can serve as a global pioneer in this regard. The findings of this study provide the necessary evidence for policymaking regarding active aging.

### Electronic supplementary material

Below is the link to the electronic supplementary material.

Supplementary Material 1

## Data Availability

The datasets generated or analyzed in the current study are not publicly available because of privacy or ethical restrictions but are available from the corresponding author upon reasonable request.

## Abbreviations

AAI: Active Aging Index
ATAA: Attitude Toward Active Aging
B: Non-standardized coefficient
β: Standardized coefficient
CI: Confidential Interval
SD: Standard Deviation
WHO: World Health Organization
